# Using Google Trends to Examine the Spatio-Temporal Incidence and Behavioral Patterns of Dengue Disease: A Case Study in Metropolitan Manila, Philippines

**DOI:** 10.3390/tropicalmed3040118

**Published:** 2018-11-11

**Authors:** Howell T. Ho, Thaddeus M. Carvajal, John Robert Bautista, Jayson Dale R. Capistrano, Katherine M. Viacrusis, Lara Fides T. Hernandez, Kozo Watanabe

**Affiliations:** 1 Office of the Vice President of Academic Affairs, Trinity University of Asia, Quezon City 1112, Philippines; larafides_hernandez@yahoo.com; 2 Department of Civil and Environmental Engineering-Faculty of Engineering, Ehime University, Matsuyama 790-8577, Japan; katviacrusis@gmail.com (K.M.V.); watanabe_kozo@cee.ehime-u.ac.jp (K.W.); 3 Biological Control Research Unit, Center for Natural Science and Environmental Research-College of Science, De La Salle University, Taft Ave Manila 1004, Philippines; jayson_capistrano@dlsu.edu.ph; 4 Biology Department-College of Science, De La Salle University, Manila 1004, Philippines; 5 Wee Kim Wee School of Communication and Information, Nanyang Technological University, Singapore 637718, Singapore; John0028@e.ntu.edu.sg; 6 Antimicrobial Resistance Surveillance Laboratory, Research Institute for Tropical Medicine, Muntinlupa City 1781, Philippines

**Keywords:** Metropolitan Manila, dengue, Google Dengue Trends (GDT), sign and symptoms

## Abstract

Dengue is a major public health concern and an economic burden in the Philippines. Despite the country’s improved dengue surveillance, it still suffers from various setbacks and needs to be complemented with alternative approaches. Previous studies have demonstrated the potential of Internet-based surveillance such as Google Dengue Trends (GDT) in supplementing current epidemiological methods for predicting future dengue outbreaks and patterns. With this, our study has two objectives: (1) assess the temporal relationship of weekly GDT and dengue incidence in Metropolitan Manila from 2009–2014; and (2) examine the health-seeking behavior based on dengue-related search queries of the population. The study collated the population statistics and reported dengue cases in Metropolitan Manila from respective government agencies to calculate the dengue incidence (DI) on a weekly basis for the entire region and annually per city. Data processing of GDT and dengue incidence was performed by conducting an ‘adjustment’ and scaling procedures, respectively, and further analyzed for correlation and cross-correlation analyses using Pearson’s correlation. The relative search volume of the term ‘dengue’ and top dengue-related search queries in Metropolitan Manila were obtained and organized from the Google Trends platform. Afterwards, a thematic analysis was employed, and word clouds were generated to examine the health behavior of the population. Results showed that weekly temporal GDT pattern are closely similar to the weekly DI pattern in Metropolitan Manila. Further analysis showed that GDT has a moderate and positive association with DI when adjusted or scaled, respectively. Cross-correlation analysis revealed a delayed effect where GDT leads DI by 1–2 weeks. Thematic analysis of dengue-related search queries indicated 5 categories namely; (a) dengue, (b) sign and symptoms of dengue, (c) treatment and prevention, (d) mosquito, and (e) other diseases. The majority of the search queries were classified in ‘signs and symptoms’ which indicate the health-seeking behavior of the population towards the disease. Therefore, GDT can be utilized to complement traditional disease surveillance methods combined with other factors that could potentially identify dengue hotspots and help in public health decisions.

## 1. Introduction

Dengue is one of the leading and most important mosquito-borne viral diseases in the world where 2.5 billion people worldwide are estimated to be at risk of contracting the disease [1]. South-East Asia has been identified to be highly vulnerable to mosquito-borne diseases [2] where dengue is highly endemic in selected countries [3,4,5,6,7,8]. Therefore, the significant health and economic burden [9] brought by dengue in the Philippines makes it a major public health concern and one of the national notifiable diseases since 1958 [10].

The National Epidemiology Centre (NEC) of the Department of Health in the Philippines has a reporting system for dengue in all its disease reporting units, such as hospitals and rural health facilities [11]. For the past decade, this traditional, health facility-based, and government-implemented surveillance system has undergone improvements, yet it still suffers from untimely reporting, aggregation, and publication of data. Due to these surveillance problems, the identification and efficiency of intervention that may prevent dengue epidemics could be limited. Many suggestions involving alternative approaches outside the virological and clinical domains (e.g., telephone triage calls, sales of over-the-counter drugs, and school/work absenteeism) have been proposed in parallel with traditional surveillance [12,13,14]. Among these non-traditional suggestions, online activity or Internet search tracking has shown the potential to complement current epidemiological methods because of its efficiency and the availability of real-time population trends [15,16,17,18,19].

Internet access and use have increased globally, including in the Philippines. The Internet, together with social media, can be used to facilitate disease surveillance, mass communication, health education, and knowledge translation and collaboration amongst healthcare providers [20]. Google Trends is an accessible Internet platform that provides geospatial and temporal patterns of search volumes for user-specified terms towards public health surveillance. The association and predictive power of Google Trends towards disease surveillance [21] have been studied extensively especially for diseases such as influenza [22], HIV [23], scarlet fever [24], malaria [25], Ebola [26], and Zika [27]. Countries like Bolivia, Brazil, India, Indonesia, Singapore, Mexico, and Venezuela have recently investigated the application of Google Trends for dengue surveillance and it has been observed to be highly correlated with the temporal pattern of dengue in a country-wide scale [16,17,28,29]. Therefore, this platform may complement in assisting real-time dengue case surveillance [16] and can be potentially extended towards predicting early dengue disease outbreaks [17]. 

Another potential advantage of using Google Trends data is it can shed light on the health-seeking behaviors of targeted populations for specific diseases by analyzing a list of search-related queries [20]. Thus, it can present significant insights into population behavior and health-related phenomena of diseases [30]. Because of its capacity to reflect important topics from search queries at a given time period, Google Trends provides valuable data on quantifying the health-seeking behavior of a specified population [31]. 

The primary aim of the study is to examine Google Trends search volume data (GDT) by revealing insights toward the pattern of dengue incidence in Metropolitan Manila, Philippines. This study has two specific objectives. The first objective assesses the temporal relationship between Google Dengue Trends (GDT) and dengue incidence in Metropolitan Manila. While such an endeavor may be deemed common due to the numerous studies with the same objective, we considered our approach in fulfilling this objective to be both methodological as well as investigative. Here we underscore the importance of data processing towards a ‘weekly’ temporal pattern, such as adjusting GDT (AdjGDT) or scaling dengue incidence (ScDI). Furthermore, we compare the temporal pattern and its association from varying processed or transformed data of GDT and dengue incidence. On the other hand, the second objective of the study explores the health-seeking behavior of users based on their dengue-related search queries. Currently, such studies do not incorporate such findings as there have been few studies that evaluated related-search queries of dengue. Therefore, investigating this matter may provide a better understanding of the population’s health-seeking behaviors regarding the disease [32].

## 2. Materials and Methods 

### 2.1. Study Area and Population Demographics

**Figure 1:**
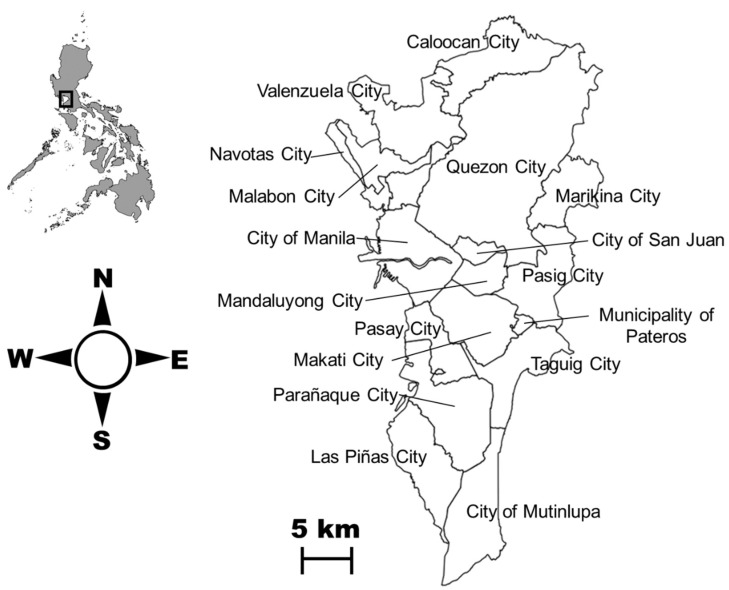
Administrative boundaries of Metropolitan Manila cities.

Metropolitan Manila is also known as the National Capital Region (NCR) of the Philippines. It is located at the eastern shore of Manila Bay in south-western Luzon, Philippines (14°50′N, 121°E) with an area of 636 km^2^. The region contains 16 cities and 1 municipality with a total population of 12.8 million [33] (Figure 1). The map layer of the administrative city boundaries of Metropolitan Manila was obtained from the Philippine Geographic Information System (GIS) Data Clearinghouse [34]. The population statistics of each city and municipality were obtained from the Philippine Statistics Authority agency [33]. The most recent Philippine population census was reported during the years 2010 and 2015. Thus, we utilized the compounded population growth rate to calculate the population of the entire region and from each city/municipality for the years 2009, 2011, 2012, 2013 and 2014 with the assumption of a fixed growth rate.

### 2.2. Data Sources

**Figure 2:**
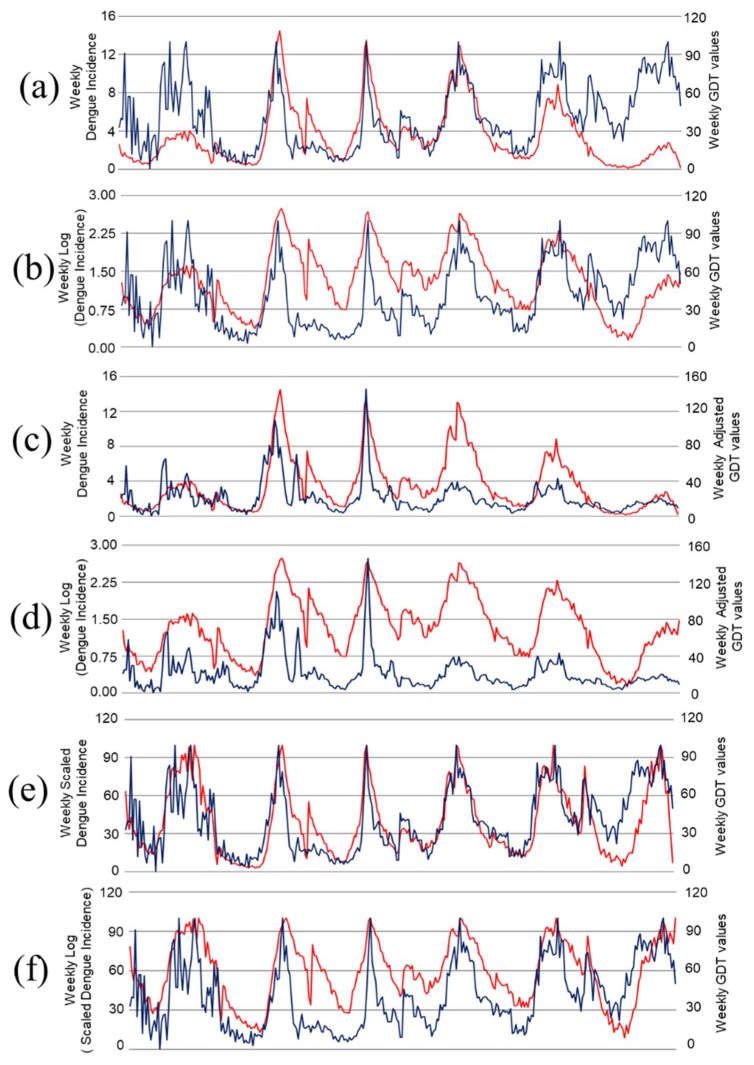
Weekly reports of dengue (red line) and Google Dengue Trends (GDT) (blue line) from 2009–2014. (**a**) Dengue incidence (DI) and GDT values; (**b**) log-transformed dengue incidence and GDT values; (**c**) dengue incidence and adjusted GDT values; (**d**) log-transformed dengue incidence and adjusted GDT values; (**e**) scaled dengue incidence and GDT values; (**f**) log-transformed scaled dengue incidence and GDT values.

Reported dengue cases of Metropolitan Manila (1st Morbidity week of 2009 until 52nd week of 2014) were obtained from the National Epidemiology Center of the Department of Health. With this, dengue incidence (DI) rate (per 1000 of the population) can be calculated by dividing the number of cases by the calculated population size for a given year, then multiplying to a factor of 1000. DI was computed in two ways: (a) annually per city (Figure 2a–f) and (b) weekly for the entire region. 

Search query data from Google Trends can be mined from their website (https://trends.google.com/trends) using the term ‘dengue’. For the purposes of this study, we abbreviate the Google Dengue Trends in the Philippines as “GDT”. This platform shows the relative search volume (RSV) which is the query share of a term (e.g., dengue) for a given time period and location. The value ranges from 0–100 are normalized from the highest query share of the term over the time series. This value also represents the ‘search interest’ index, where a term value of 100 is denoted as the peak popularity while a value of 50 represents exactly half of the popularity. On the other hand, a term with a value of zero means insufficient data. Additional data displayed are the cities during the queried time period in Metropolitan Manila that search for the term ‘dengue’. GDT also displays words and phrases referred to as ‘related queries’ of dengue. These queries are divided into (a) top and (b) rising categories. The top category refers to the most popular search queries and contains score values (0–100). While, the rising category refers to queries with the biggest increase in search frequency since the last time period. A select number of search queries can be labelled as ‘breakout’ referring to a tremendous increase at that given time. 

### 2.3. Data Processing and Analysis

Initially, we identified four dengue data types and two GDT data types for association analysis using Pearson correlation. The use of Pearson’s correlation follows the choice of previous studies [16,27,28] that correlated GDT and dengue and, in addition, no controls for correlated errors were used. The first GDT data type refers to the generated weekly value for a yearly period. It should be emphasized that GDT values are relative and can change depending on the specified time range used during the search query. For instance, the GDT value for a specific week using a one-year query would not be necessarily the same when a two-year query is used. This is because a displayed GDT value is highly dependent on the highest query volume for a given time period. Moreover, GDT has a limit or restriction in displaying weekly values. It can only generate weekly values up to a maximum time period of 4 years and further than that will result in generating monthly values. If one does not realize how these values are calculated and displayed by Google Trends, researchers opt to ‘stitch’ together yearly GDT values, which is inappropriate. 

To resolve this issue, a GDT adjustment procedure proposed by Risteski and Davcev [35,36] was implemented since the study has a 6-year time period. To employ the GDT adjustment, we obtained the monthly-level GDT query value of dengue for the entire period of 2009–2014 and then collected GDT weekly-level query value per year of 2009–2014. Afterwards, we multiplied each weekly-level value by the monthly-level query result and divided it by the monthly-level average from the weekly data. This adjustment procedure accounts for differences in the relative prevalence of searches over time in the stacked weekly-level data. Thus, the second GDT data type is the weekly adjusted value (AdjGDT). On the other hand, the first dengue data type is the calculated weekly dengue incidence rate (DI) while its log transformed (LogDI) is the second dengue data type. Log transformation was performed in order to reduce the skewing of the data and destabilize the variance. 

Since GDT generates a relative value based on the highest query volume for a given time period, we followed this type of calculation to DI in order for it to be in the same unit of magnitude as GDT. This is achieved by dividing the observed value to the maximum observed value of a given year (2009–2014). Therefore, the third and fourth dengue data types are referred to as the scaled DI (ScDI) and its log transformed (LogScDI), respectively. Additionally, cross-correlation analysis was performed from 0–25 week lags across all data types. Computations were done using the stats package of the R program version 3.3.5 [37]. We analyzed the GDT values in identified cities over each time period with respect to each city’s dengue incidence by Pearson’s correlation, and heat maps were generated using ArcGIS version 10.2 [38]. 

The top and rising dengue-related search queries for each period were collected and organized accordingly. Thematic analysis was performed on the search queries listed in the top query category. To quantify the categorized themes per year, the percentage value was used by obtaining the sum of GDT values for each year and category and then dividing it to the maximum GDT summation value of a particular year. Word clouds of the top search queries in each period were created using WordArt (www.wordart.com). Previous studies have used this web application to create customized word clouds wherein the size of the words or phrases can be adjusted based on their frequency [39,40]. However, instead of frequency, the size of each search query was based on the ‘interest over time’ value provided by Google Trends. Simply put, bigger words in the word cloud mean high search query value, and in turn, mean high interest over time. In this study, the word cloud was patterned using the map of Metropolitan Manila. 

## 3. Results

### 3.1. Association of Google Dengue Trends (GDT) and Dengue Incidence (DI) 

Figure 2 shows the temporal pattern of all dengue and GDT data types. It can be observed that the GDT trend (Figure 2a,b) follows the seasonal dengue pattern of Metropolitan Manila. However, 2009, 2013, and 2014 GDT trend pattern do not closely match the expected dengue incidence (DI) pattern except when it is transformed into its logarithmic function (LogDI). Further analysis demonstrated that the association of observed weekly values of DI were moderately associated (*r* = 0.405) to the weekly GDT values and the association between GDT and LogDI was slightly lower (*r* = 0.394) (Table 1). 

As per Risteski and Davcev’s [35,36] recommendations, the weekly GDT values (AdjGDT) were adjusted accordingly. It can be observed that the AdjGDT values still follow the seasonal dengue pattern except for the periods of 2012 and 2013 and the trend pattern of the AdjGDT does not conform to the temporal pattern of LogDI (Figure 2c,d). The association of the AdjGDT values showed higher correlation coefficients for DI (*r* = 0.662) and log transformed DI (*r* = 0.597) as compared to unadjusted GDT (Table 1). Interestingly, when the DI was scaled accordingly (ScDI and LogScDI) and plotted with the unadjusted GDT values, the temporal trends were nearly similar to each other (Figure 2e,f). Moreover, the association of GDT to ScDI (*r* = 0.747) and LogScDI (*r* = 0.660) were higher as compared to the association of DI from unadjusted and adjusted GDT (Table 1). Lastly, when AdjGDT and ScDI were plotted, the temporal patterns did not closely match each other (data not shown). These observed patterns are the same between DI and GDT (Figure 2a,b). However, correlations of AdjGDT with ScDI (*r* = 0.529) and LogScDI (*r* = 0.470) were higher as compared to unadjusted and unscaled variables.

In addition, Table 1 shows the cross-correlation analysis (lag effects) (Table S1) of GDT to dengue incidence. The majority of all cross-correlation analyses revealed the highest lag association to be at lags 1 and 2 weeks. This can be visually observed in selected high peaks of both dengue and GDT in Figure 2. For example, in Figure 2e (ScDI and GDT), prior to the highest dengue incidence peak in 2010, a high GDT peak occurred a week before. 

### 3.2. Spatial Pattern of GDT and Related Queries for Dengue

**Figure 3:**
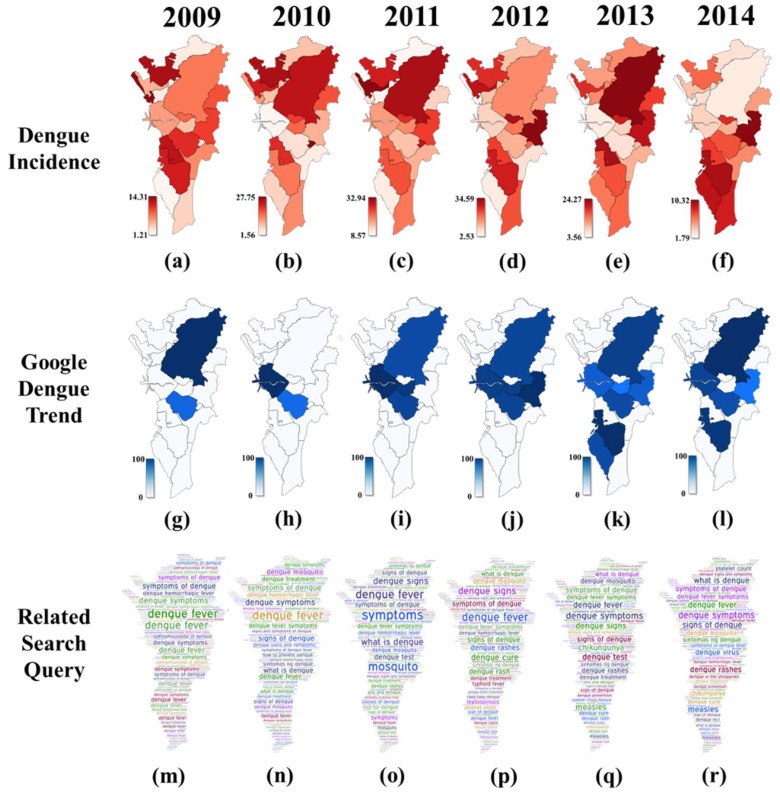
City level spatio-temporal pattern of dengue incidence (**a**–**f**) and Google Dengue Trends (**g**–**l**). Temporal word clouds maps of dengue-related search query in Metropolitan Manila (**m**–**r**).

Figure 3 shows the GDT values for Metropolitan Manila cities from 2009–2014. It is noteworthy that the cities with GDT increased from two in 2009 to seven in 2013. Makati consistently had GDT data for the entire 6-year period. This is followed by cities, such as Quezon (5 years), Manila (5 years), Pasig (3 years), Mandaluyong (3 years), Paranaque (2 years), and Las Piñas (1 year). Comparing the spatial maps of dengue incidence (Figure 3a–f) and GDT values per city showed no discernable pattern revealing high dengue incidence in a city with high GDT value. Further analysis showed a very low and non-significant relationship (*r* = 0.223, *p* = 0.283).

Table 2 shows that the top search queries from 2009 to 2014 were categorized under five groups. These were (1) dengue, (2) signs and symptoms, (3) treatment and prevention, (4) mosquito, and (5) other diseases. Based on the GDT percentages across each category and year, many of the listed top queries were related to dengue and its symptoms. Search queries under ‘dengue’ were more popular in 2009. Search queries under this group consisted of phrases related to dengue’s etiology (e.g., causes of dengue and dengue virus) and alternative names (e.g., dengue hemorrhagic fever and dengue fever).

Over the six-year period, search queries related to ‘signs and symptoms’ were more visually pronounced in each word cloud (Figure 3m–r) considering that more users searched for terms related to dengue signs and symptoms from 2010 to 2014. While some search queries were aimed at retrieving information on dengue’s signs and symptoms using search queries, such as ‘symptoms’ and ‘dengue signs and symptoms’, other search queries denote specific signs and symptoms such as ‘dengue rash’ and ‘platelet count’.

Some search queries were also about dengue ‘treatment and prevention’. A few notable search queries under this group were: (1) ‘ncp for dengue’, which stands for Nursing Care Plan (a reflection of the popularity of nursing as a tertiary-level course in the Philippines); (2) ‘dengue NS1’ which concerns the dengue NS1 antigen test; and (3) ‘tawa-tawa dengue’ which refers to *Euphorbia hirta* Linn, a herbal medicine believed to help treatment of dengue.

Another category involved ‘mosquito’ which includes search queries such as ‘dengue mosquito’ and ‘mosquito’. Lastly, the ‘other diseases’ category showed disease-specific search queries that appeared only in 2012 to 2014. ‘Leptospirosis’ and ‘typhoid fever’ were relevant to dengue since these occur during rainy seasons in which dengue cases tend to be more prevalent [41]. ‘Measles’ also appeared since flooding during the rainy seasons can lead to displacement of citizens in evacuation centers where measles tends to spread rapidly [42]. ‘Chikungunya’ was also a relevant search query due to its similarity to dengue and the considerable media attention it received in 2013 because of occasional outbreaks [42].

### 3.3. Rising and Breakout Search Queries

A rising search query is defined by an accompanying percentage which reflects the search query’s growth in volume in a given period with respect to the preceding period [43] while breakout search query is defined by a tremendous increase in percentage. Higher percentages indicate that the search query is trending in Google [44,45]. Table 3 enumerates the list of rising and breakout search queries for each period.

Results showed that the listed rising search queries per year revolved around the five previously-mentioned thematic categories. A majority of the search queries were related to ‘signs and symptoms’. Although most of the search terms had a rising search percentage of 40% to 1550%, there were some that achieved breakout—search queries that grew by more than 5000% [45]. The breakout search queries from 2011 reflect finding more information about the name (e.g., ‘dengue fever syndrome’), symptoms (‘symptoms of dengue’ and ‘*mga sintomas* ng dengue’), and cases (‘dengue cases in the Philippines’) of dengue. Interestingly, while the search query ‘symbianize’ might be irrelevant, it achieved breakout since it is a search query related to a popular Philippine forum website where users go to search and provide health information about dengue. Another interesting breakout search query is ‘Michael V dengue’ in 2013. This situation reflects the event when Michael V, a well-known Filipino celebrity, announced on Twitter that he was diagnosed with dengue [46].

## 4. Discussion

### 4.1. Pattern of GDT and Dengue Incidence

The study determined the association of GDT to dengue incidence where it emphasized the importance of adjusting GDT (AdjGDT) or scaling dengue incidence (ScDI) accordingly for correlation analysis. For instance, in Table 1, the association was improved when GDT was adjusted or DI was scaled in accordance to how GDT is calculated. It also showed that the adjusted GDT captured the similar temporal trend of DI (Figure 2c), resulting in a better estimate of the association. Furthermore, if dengue incidence was scaled accordingly (Figure 2e), it showed that it can nearly capture the unadjusted GDT trend which also resulted to an improved correlation coefficient. Therefore, the processing methods clearly show how the test statistic is considerably affected which can undermine the nature of its actual association. 

If one is not familiar with either the limitations of the GDT value, ‘stitching’ of weekly values per year may erroneously lead to an inappropriate interpretation of its association (e.g., GDT and DI). Moreover, scaling of DI was simple in order to demonstrate the relative nature of the GDT value, and by standardizing it in the same unit of magnitude would generate a better and improved association. Previous dengue studies that assessed the association with GDT used a monthly time scale, therefore, an adjustment procedure is unnecessary. In most dengue-endemic countries such as the Philippines, health agencies implement and follow a weekly reporting of the said disease [47]. Although it is possible and easier to aggregate the weekly number of cases to generate a monthly value, investigating and utilizing a weekly temporal scale are more informative and useful in future applications. 

Likewise, we also investigated on how log-transformation of dengue incidence (LogDI) can affect the association with GDT. When we compare the correlation coefficients of DI and LogDI with adjusted or unadjusted GDT, DI showed a slightly better correlation estimate than LogDI. It should be emphasized that while previous studies utilized either DI [17,27] or total number of dengue cases [28,48] our study stresses that choosing a certain dengue data type could affect the association with GDT. Therefore, future researchers investigating this endeavor should consider not only what GDT data type to use appropriately but also the dengue data type. 

In Table 1, the temporal correlation between GDT and dengue was revealed to be moderately associated (*r* = 0.394 to 0.747). This is in contrast with previous studies [27,28] that yielded high correlation coefficients (e.g., 0.8–0.9). However, these studies examined the association with GDT in a nation- or country-wide scale as compared to the present study that focused only to one region in the Philippines. There is only one study that investigated the association of GDT and dengue in a regional scale where the correlation among different regions varies substantially with a high correlation coefficient resulting from regions with higher dengue incidence [27]. We infer that other factors such as the climate play a role in the temporal pattern of dengue incidence in Metropolitan Manila as suggested by a recent study [49]. Therefore, it is further recommended and worthwhile to investigate the incorporation of GDT with climate-based models in predicting future dengue risk from novel statistical approaches [27,49]. 

Further analysis using cross-correlation results revealed that GDT has a delayed effect (1–2 weeks) to the reported dengue incidence. We hypothesized that this delayed effect may stem from the users’ self-diagnosis of their medical condition before going to the hospital for possible admission and management of the disease. Given that we are in the information age, it is more likely that users are seeking health information through the Internet, thus having an idea about their illness before consulting a doctor. This corroborates with our thematic analysis where majority of search queries of dengue refer to its ‘signs and symptoms’. It was claimed that an increase of health-seeking behavior from Internet users would pertain the surrounding environment being affected by the disease [28]. Therefore, GDT could complement as a real-time disease monitoring tool [48]. However, this approach has its limitations and drawbacks as exemplified when Google Trends was assessed during the influenza pandemic of 2009, the 2012/2013 flu epidemic season in the US and, the Ebola pandemic in Africa [47,50]. These studies outlined that the Google Trends platform is limited to the proportion of the population who use the Internet to obtain health-related information [47], intense media coverage [50,51,52] and algorithm dynamics [51,52,53]. Moreover, the application of GDT may only be suited to areas with high dengue incidence [29]. Since the study was only limited in investigating one region in the Philippines, our interpretation cannot be the same in other regions with a large proportion of users situated in rural areas where Internet access is limited. 

In general, publicly available GDT data may be a promising tool for dengue surveillance but there should be caution when using it as the primary basis for public health decisions. Although previous studies [17,21] and the results of our analysis showed the temporal relatedness of GDT and dengue incidence, relying only on search query data for making future public health decisions on dengue may trigger unnecessary panic from the public [48]. On the contrary, results from GDT can help to identify hotspots where further dengue investigations can be conducted. This is in line with the concept of ‘now-casting’ where potential locations for dengue cases can be predicted at present (through real-time monitoring of GDT data) rather than in the future to prevent outbreaks [16]. While countries with underdeveloped disease surveillance systems can greatly benefit from real-time monitoring of GDT data to supplement traditional epidemiologic investigations when identifying hotspots [17], caution should still be made considering that not all actual hotspots can be identified due to variations in Internet access [16]. Overall, GDT’s capability as an early warning signal for outbreaks can only be attained if it can be combined with meteorological, environmental, social, and entomological factors to produce a robust dengue prediction model [48,54]. Therefore, more work is needed to establish the reliability and real-world applicability of GDT results for dengue surveillance and control [21].

### 4.2. Search Query Behavior towards Dengue

The results showed that most of the search query activity for dengue came from the cities of Makati, Quezon, and Manila. These cities also consistently showed high GDT values across all time periods. These three cities share common characteristics that resulted to such an outcome. One of the characteristics could be the cities’ high population density that contributes to the presence of a high proportion of Internet users. Previous works in the US suggest that high population density correlates with more Internet users [52,55]. Another characteristic would be the presence of major business districts, hospitals, educational institutions and recreational activities (e.g., shopping malls) in these cities which indicates rapid urbanization. A study from 28 Asian countries, including the Philippines, showed that urbanization is related to higher Internet penetration [56]. As of January 2018, the Internet penetration rate in the Philippines is 63% which translates to 67 million users [57], the majority of whom are residents of urban areas such as Metropolitan Manila. In general, people living in urban areas with high population density tend to have more Internet access and this facilitates greater health seeking on the Internet. In line with this, the aforementioned characteristics of these cities with GDT values could partially explain why the study did not find a significant spatial relationship with DI at the city level. It should be emphasized that each identified dengue case would use the patient’s residential location to report in the national health agency. Therefore, we hypothesize that search activity in dengue may likely occur in a person’s work or school location (e.g., Makati, Quezon, or Manila) rather than in their residence (e.g., San Juan). Search queries performed by users would not particularly coincide with their place of residence since this highly urbanized region would promote frequent mobilization or traveling from one city to the other. Moreover, Internet access in these highly-developed cities may be considerably better than their residence due to the occurrence business districts and recreational activities (e.g., shopping malls).

Based on the thematic analysis of the search queries, it can be inferred that users were mostly performing health-seeking on dengue. Specifically, ‘top’, ‘rising’, and ‘breakout’ search queries mostly consist of search queries that reflect seeking information about dengue including its signs and symptoms. This is somewhat expected considering that the Internet acts an enabler for people to search for health information [58]. At the same time, the findings reflect the knowledge gap hypothesis [59] wherein people from an urban area, like Metropolitan Manila (regardless of socioeconomic background), can reduce their knowledge gaps about health-related queries (dengue) through the Internet. 

While the findings indicate that the Internet acts as an empowering technology for people to gain more knowledge about dengue, a consequence of health-seeking over the Internet is self-diagnoses of dengue without proper medical consultation [60,61]. Such a situation might lead to ‘cyberchondriasis’ where people start to develop health anxiety [62]. Scholars have advised that searching for disease-specific search queries may not correspond to true illness [15]. Considering that some of dengue’s signs and symptoms might overlap with other diseases, such as chikungunya and measles, the values reflected in Google Trends might not fully indicate disease-specific occurrence and might just reflect greater health anxiety among people. 

Another way of interpreting the findings is through the lens of the agenda-setting theory [63]. The theory indicates that the media (news outlets) influence the placing of importance upon topics of the public agenda. This could explain some of the breakout search queries since it might be a result of high media coverage of an incident that reflect the search query. For instance, media reports of increasing dengue cases in Metropolitan Manila and other regions in 2011 [64] might have led people to perform more search queries related to dengue signs and symptoms than in 2010. Moreover, the proliferation of news in 2013 related to the dengue diagnosis of Michael V, a Filipino celebrity [46], might have spiked interest to search for ‘Michael V dengue’ on Google during that year.

## 5. Conclusions

The study demonstrated the temporal relationship between the weekly patterns of GDT and dengue. It was able to reveal the necessary adjustment or scaling procedures in order to capture the appropriate temporal trend and produce improved correlation coefficients between the two values. The study was also able to demonstrate a delayed (lag) effect of GDT towards dengue incidence which has the potential to be utilized in detecting future disease outbreaks and patterns. However, the study found a non-significant spatial relationship between GDT and DI at the city level which suggests that dengue search activity would likely occur in different locations apart from the user’s residence. 

Although Google Trends is limited to the proportion of Internet users and may possibly be only suitable in areas with high disease incidence and Internet penetration, it can be used to assist traditional disease surveillance. Moreover, while it is unclear how these limitations can be addressed, it can be used as a core component for future studies regarding the utilization of Google Trends for disease surveillance. While GDT data can be used to supplement traditional surveillance methods in a developing country like the Philippines, it should be combined with other factors (e.g., meteorological, environmental, social, and entomological) to improve its applicability for public health decisions.

The study also revealed health seeking behaviors of the population by evaluating dengue-related search queries in Metropolitan Manila from 2009–2014. Thematic analyses revealed five categories namely; (a) dengue, (b) sign and symptoms of dengue, (c) treatment and prevention, (d) mosquito and (e) other diseases. Further analysis showed that majority of the search activity pertains to the ‘signs and symptoms’ of dengue. These findings indicate how the Internet acts as an empowering technology in gaining knowledge about dengue. However, users might also utilize this technology in conducting self-diagnoses without proper medical consultation. In addition to this, the high dengue search activity of the population may be attributed to the influence of media which could have led the population to perform more search queries related to dengue’s signs and symptoms. 

## Figures and Tables

**Table 1:** Correlation analysis of dengue and Google Dengue Trends (GDT) and its delayed effects (R^2^).

Dengue Values	Google Dengue Trend Values			
	Google Dengue Trend		Adjusted Google Dengue Trend	
	*r*	*lag week* (R^2^)	*r*	*lag week* (R^2^)
Dengue Incidence	0.405	1 (*0.166*)	0.662	1 (*0.465*)
Log (Dengue Incidence)	0.394	1 (*0.162*)	0.597	2 (*0.385*)
Scaled Dengue Incidence	0.747	1 (*0.570*)	0.529	2 (*0.305*)
Log (Scaled Dengue Incidence)	0.576	1 (*0.342*)	0.470	2 (*0.245*)

**Table 2:** Thematic categories of top search queries related to dengue from 2009–2014 in Metropolitan Manila. Values from 2009–2014 are percentages (%) based on the overall GDT values of each category per year.

Category.	2009	2010	2011	2012	2013	2014	Search Words
**Dengue**	100	70	52	50	29	37	what is dengue, *ano ang* dengue, causes of dengue, dengue virus, dengue hemorrhagic fever, dengue fever
**Signs and Symptoms**	84	100	100	100	100	100	symptoms, dengue symptoms, dengue symptoms Philippines, symptoms of dengue, dengue fever symptoms, dengue signs, signs of dengue, sign of dengue, signs of dengue fever, dengue signs and symptoms, signs and symptoms of dengue, sign and symptoms of dengue, *sintomas* ng dengue, symptoms of dengue fever, dengue symptoms in children, dengue rashes, dengue rash, platelet count, platelet count dengue, pathophysiology of dengue
**Treatment and Prevention**		7	14	16	10	12	dengue cure, dengue treatment, dengue fever treatment, how to prevent dengue, dengue prevention, ncp for dengue, dengue test, dengue ns1, *tawa tawa* dengue
**Mosquito**		5	13	7	4	4	dengue mosquito, mosquito
**Other Diseases**				5	6	5	leptospirosis, typhoid fever, measles, chikungunya

**Table 3:** Rising and breakout search queries related to dengue from 2009–2014 in Metropolitan Manila.

Year	Search Query	%
2009	symptoms of dengue	90%
2010	dengue signs and symptoms	300%
	signs of dengue	170%
	signs and symptoms of dengue	170%
	dengue treatment	60%
2011	dengue fever syndrome	***B***
	symtoms of dengue	***B***
	*mga sintomas* ng dengue	***B***
	symbianize	***B***
	dengue symptoms in adults	***B***
	dengue cases in the philippines	***B***
	dengue symptoms in children	500%
	ncp for dengue	250%
	dengue test	180%
	mosquito	170%
	dengue rashes	160%
	dengue mosquito	110%
	symptoms	100%
	causes of dengue	100%
	*sintomas* ng dengue	70%
	dengue prevention	70%
	what is dengue	60%
	symptoms of dengue	50%
	signs of dengue	40%
2012	signs of pregnancy	***B***
	leptospirosis	900%
	normal platelet count	400%
	dengue rash	200%
	cause of dengue	150%
	dengue rashes	110%
	typhoid fever	100%
	dengue symptoms philippines	100%
	*mga sintomas* ng dengue	90%
	dengue cure	80%
	dengue prevention	70%
	dengue fever treatment	50%
	symptoms of dengue	50%
	symptoms of dengue in children	50%
	dengue signs and symptoms	40%
2013	michael v dengue	***B***
	chikungunya	1550%
	chikungunya symptoms	700%
	*ano ang sintomas* ng dengue	130%
	signs and symptoms of dengue	120%
	measles	120%
	signs of dengue fever	110%
	dengue fever stages	100%
	dengue ns1	90%
	symtoms of dengue	70%
	*sintomas* ng dengue	60%
	sign of dengue	60%
	signs of dengue	50%
2014	cause of dengue	350%
	symptoms of pregnancy	120%
	dengue ns1	60%
	what is dengue	50%
	dengue in the philippines	40%

***B***—Breakout.

## Supplementary Materials

The following are available online at http://www.mdpi.com/2414-6366/3/4/118/s1, Table S1: Cross-correlation analysis (R^2^) of Dengue incidence and Google Dengue Trends.
