# Supplementary material for: Using Google Trends to Examine the Spatio-Temporal Incidence and Behavioral Patterns of Dengue Disease: A Case Study in Metropolitan Manila, Philippines
Source: Trop Med Infect Dis. 2018 Nov 11;3(4):118. doi: 10.3390/tropicalmed3040118 (PMC6306840; doi:10.3390/tropicalmed3040118)
Supplement: Supplementary file 1 [file tropicalmed-03-00118-s001.pdf]

Tropical Medicine and Infectious Disease

MDPI

Table S1. Cross-correlation analysis (R^2) of Dengue incidence and Google Dengue Trends.

Lag Weeks	GDT				AdjGDT			
	**DI**	**LogDI**	**AdjDI**	**AdjLogDI**	**DI**	**LogDI**	**AdjDI**	**AdjLogDI**
Lag Week 0	0.164	0.156	0.558	0.332	0.438	0.356	0.280	0.221
Lag Week 1	0.166	0.162	0.570	0.342	0.465	0.382	0.305	0.242
Lag Week 2	0.152	0.153	0.537	0.331	0.458	0.385	0.305	0.245
Lag Week 3	0.130	0.141	0.480	0.312	0.428	0.369	0.282	0.236
Lag Week 4	0.106	0.125	0.428	0.290	0.383	0.340	0.252	0.218
Lag Week 5	0.077	0.100	0.354	0.257	0.338	0.307	0.212	0.195
Lag Week 6	0.051	0.075	0.283	0.215	0.299	0.282	0.177	0.178
Lag Week 7	0.028	0.051	0.213	0.176	0.248	0.248	0.138	0.156
Lag Week 8	0.012	0.032	0.164	0.139	0.188	0.203	0.098	0.127
Lag Week 9	0.002	0.016	0.112	0.105	0.131	0.156	0.061	0.098
Lag Week 10	0.001	0.004	0.065	0.068	0.082	0.110	0.030	0.067
Lag Week 11	0.008	0.000	0.036	0.038	0.044	0.069	0.010	0.040
Lag Week 12	0.023	0.004	0.014	0.019	0.017	0.038	0.001	0.020
Lag Week 13	0.042	0.014	0.003	0.007	0.004	0.017	0.002	0.007
Lag Week 14	0.065	0.028	0.000	0.002	0.000	0.005	0.010	0.002
Lag Week 15	0.089	0.046	0.005	0.000	0.002	0.001	0.023	0.000
Lag Week 16	0.109	0.061	0.010	0.002	0.009	0.000	0.035	0.001
Lag Week 17	0.125	0.074	0.019	0.005	0.014	0.002	0.045	0.004
Lag Week 18	0.144	0.091	0.032	0.009	0.019	0.003	0.058	0.005
Lag Week 19	0.165	0.114	0.049	0.017	0.025	0.005	0.070	0.006
Lag Week 20	0.180	0.133	0.061	0.026	0.028	0.007	0.075	0.007
Lag Week 21	0.183	0.145	0.065	0.035	0.028	0.008	0.077	0.008
Lag Week 22	0.197	0.168	0.080	0.048	0.034	0.012	0.088	0.011
Lag Week 23	0.211	0.200	0.093	0.072	0.036	0.016	0.093	0.016
Lag Week 24	0.214	0.219	0.096	0.090	0.036	0.018	0.094	0.019
Lag Week 25	0.221	0.236	0.103	0.102	0.038	0.021	0.100	0.023

GDT = Google Dengue Trends; AdjGDT = Adjusted Google Dengue Trends; DI = Dengue Incidence; LogDI = Logarithm Transformed Dengue Incidence; AdjDI = Adjusted Dengue Incidence; AdjLogDI = Adjusted Logarithm Transformed Dengue Incidence.
